# Caregivers of people with Alzheimer’s Disease in Puerto Rico 2021 ‐ 2022: A panoramic view of their epidemiological profile, health status and quality of life

**DOI:** 10.1002/alz.087391

**Published:** 2025-01-09

**Authors:** Alex Cabrera, Marcos E. Felici, Marianne Cartagena

**Affiliations:** ^1^ Puerto Rico Department of Health, San Juan, Puerto Rico Puerto Rico

## Abstract

**Background:**

Alzheimer’s Disease (AD) is the fourth leading cause of death in Puerto Rico (PR), with an estimated prevalence of 14%. Caregivers are an essential part in the management and care of people with AD. Providing care for a person with Alzheimer can change over time and can become a challenge for the caregivers. For this reason, self‐care is one of the most important things that a caregiver must do. The main objective of this research is to perform an epidemiological profile of caregivers for people with Alzheimer in PR and analyze the health status and quality of life variables collected by the PR Behavioral Risk Factor Surveillance System (PRBRFSS).

**Method:**

A descriptive cross‐sectional study was carried out using the PRBRFSS data for 2021‐2022. Univariate analysis was performed to obtain an epidemiological profile of caregivers for people with Alzheimer in Puerto Rico, as well as to analyze health status and quality of life variables.

**Result:**

For 2021‐2022, according to the PRBRFSS, a total of 318,924 people in Puerto Rico identified themselves as caregivers. Among these, 16.9% reported being a caregiver of a person with Alzheimer. Approximately 23.0% of the caregivers for people with AD were in the 55‐64 age group, 63.9% were women, 41.4% were married, 70.6% had education above high school, and 47.2% had an annual income of $25,000 or more. In terms of health and quality of life, 23.1% reported having bad or poor health, 23.6% reported having depression, and 60.7% reported at least one comorbidity. Likewise, 24.4% reported difficulty seeing, 23.1% walking or ascending stairs, 18.6% concentrating or remembering, 11.0% performing tasks alone, 8.0% hearing, and 6.5% dressing or taking a bath.

**Conclusion:**

Caregiving for a person with Alzheimer becomes more challenging over time due to the evolution of symptoms and the level of care needed. For this reason, caregivers need to maintain good health and reduce the risk of burnout. This research demonstrates the importance of continuing to create programs and support groups for caregivers of people with Alzheimer to encourage self‐care and improve their health and quality of life.

